# Family function, anxiety and depression in adults with disabilities: a network analysis

**DOI:** 10.3389/fpubh.2023.1181203

**Published:** 2023-10-31

**Authors:** Bin Wang, Dongling Yuan, Xiao Zhong, Fan Yang, Haojie Fu

**Affiliations:** ^1^Research Center of Psychosocial Service and Crisis Intervention, Southwest University of Science and Technology, Mianyang, China; ^2^Medical Psychological Center, The Second Xiangya Hospital, Central South University, Changsha, China; ^3^Department of Psychology, Beijing Sport University, Beijing, China; ^4^Guangan Psychiatric Hospital, Guangan, China; ^5^Shanghai Research Institute for Intelligent Autonomous Systems, Tongji University, Shanghai, China

**Keywords:** network analysis, family function, anxiety, depression, adults with disabilities

## Abstract

**Background:**

The prevalence of family dysfunction, anxiety and depression is high in people with disabilities due to long-term activity constraints and social difficulties. Recently, although studies have attempted to provide guidance for family therapy by focusing on the relationship between family function and negative emotions, the specific effects of improved family function during family therapy on alleviation of anxiety and depressive symptoms have been obscured. Thus, this study attempted to elucidate the impact of specific family functioning on specific symptoms of anxiety and depression through network analysis.

**Methods:**

Family APGAR Index Questionnaire (APGAR), Generalized Anxiety Scale (GAD-7), and Patient Health Questionnaire Depression Scale (PHQ-9) were used to survey 897 adults with disabilities in Sichuan Province. Meanwhile, network analysis for studying the relationship between anxiety, depression and family functioning among the disabled via R software.

**Results:**

The network analysis showed that (1) Nodes PHQ4 (“Energy”), APGAR3 (“Growth”), GAD1 (“Nervousness”) and GAD4 (“Relaxing Trouble”) were central nodes in the network model; (2) Bridge nodes linking family function, anxiety and depressive symptoms in the sample were PHQ9 (“Suicide ideation”), PHQ6 (“Worthlessness”), GAD1 (“Nervousness”) and GAD5 (“Restlessness”); (3) The node APGAR5 (“Resolve”) directly connects the bridge symptoms PHQ9 (“Suicide ideation”) and PHQ8 (“Motor”).

**Conclusion:**

This study suggests that therapists could target the resolve of family members during family therapy to reduce suicidal ideation and enhance the level of activity of people with disabilities, thereby improving the network of anxiety and depression symptoms and alleviating negative emotions of people with disabilities.

## 1. Introduction

Disability results from the interaction between individuals with a health condition, such as cerebral palsy, Down syndrome and depression, with personal and environmental factors including negative attitudes, inaccessible transportation and public buildings, and limited social support.^1^ According to the results of the second national sampling survey of people with disabilities in China, there were 82.96 million people with disabilities, accounting for 6.34% of China's total population (1). Studies have shown that with long-term activity constraints and social difficulties, people with disabilities are prone to psychological disorders, mainly manifested as severe anxiety and depression (1, 2). A large cohort study showed that 67% of depressed patients have a current comorbid anxiety disorder, while 63% of patients with an anxiety disorder also have depression (3). Moreover, a recent study found that the prevalence of depression combined with anxiety in middle-aged and older adults was 6.67%, with a higher prevalence among middle-aged and older adults with disabilities (4). However, studies have also found that psychological interventions, especially family therapy focused on improving family function, can effectively reduce anxiety and depression (5, 6).

Family function refers to creating a good material and spiritual environment for individual physical and psychological development to meet each family member's growth and socialization needs, including adaptation, partnership, growth, affection and resolution (7). The theory of family functional systems suggests that if families fail to achieve the basic function of the family, various clinical problems will arise among family members (8). That is, the lack of open communication and interaction between family members leads to alienation from each other in dysfunctional families, which may lead to the accumulation of negative emotions that affect the social adjustment of family members and eventually induce psychological disorders such as anxiety and depression (2). Abramson's hopelessness theory (9) also suggests that if external stimuli (e.g., discrimination, family function) are negative, individuals tend to draw negative conclusions about themselves, leading to hopelessness and depression. Meanwhile, the study also indicated that people with disabilities are prone to family dysfunction due to their own disabilities (2), while poor family functioning tends to trigger psychological disorders such as anxiety and depression in family members (10). Notably, however, these studies only looked at the relationship between family function and overall anxiety and depression. Namely, the level of family function was negatively correlated with individual anxiety and depression levels (10). This ignores the effect of specific dimensions of family function on anxiety and depressive symptoms, thus obscuring the specific effects of improved family function during family therapy on the alleviation of anxiety and depressive symptoms. In addition, the study of emotional disorders also revealed that affective symptoms form a network of direct interactions (11). In addition, researchers considered family function, anxiety, and depression as interrelated systems, with changes in each component of the system triggering changes in others (10). Thus, based on the above, network analysis was essential to explore the relationship between family function, anxiety and depression among adults with disabilities.

In contrast to traditional perspectives, network analysis treats symptoms as components of mental disorders (12, 13), whereas the emergence and development of mental disorders are thought to be caused by strong causal interactions between symptoms (12, 14). Therefore, an important goal of the network analysis approach is to identify the most influential symptoms in the underlying symptom network, which are defined as highly centralized symptoms. Moreover, core symptoms may be more likely to activate other symptoms in the network, driving the development of psychiatric disorders (13, 15).

Comorbidity of anxiety and depression is widespread, so many studies have been conducted to explore the comorbidities of depression and anxiety based on network analysis. For example, Makhubela (16) and Briganti et al. (17) also showed that anhedonia and low energy were the core symptoms, while Beard et al. (18) found in a psychiatric sample that “depressed mood” and “worry” were the most central symptoms in the depression and anxiety network. Zhang et al. (19) also found in the older adult that “feeling sad” and “trouble relaxing” were central symptoms in the depression and anxiety symptom network. These suggest that there is a strong correlation between depression symptoms and anxiety networks, but also that core symptoms vary across groups. While for people with disabilities, extreme underlying psychopathological vulnerability such as social exclusion and poor coping strategies (20) contribute to an increased risk of mood disorders, there may be heterogeneity in clinical manifestations of mood disorder symptoms. In addition, the general strain theory also suggests that a lack of interpersonal relationships is prone to induce nervousness, while family bonds are the most important interpersonal relationship for people with disabilities (21). This may be why positive family interaction can effectively alleviate negative emotions and reduce psychological disorders in people with disabilities. Moreover, the study has also shown that good family function is effective in improving anxiety and depression levels in people with disabilities (10). More importantly, fewer studies have focused on family functioning, anxiety and depressive symptom networks for people with disabilities. Therefore, by exploring the dimensions of family function on which specific symptoms of depression and anxiety affect the entire depression and anxiety network, it may be important to improve the effectiveness of family therapy focused on improving family function.

In summary, this study proposed the use of network analysis to clarify the complex relationship between family function, anxiety and depression symptoms in people with disabilities mainly based on the theory of family functional systems, thus precisely identifying the core dimensions of family function and the maintenance mechanisms of anxiety and depression symptoms in people with disabilities, and thus providing a foundation for precise family therapy.

## 2. Methods

### 2.1. Participants and procedure

The researchers conducted an online questionnaire survey from February 28 to March 26, 2022 through “Questionnaire Star” among adults with disabilities who met the following selection criteria: (1) age 18 or older; (2) registered adults with disabilities of the China Disabled Persons' Federation; (3) comprehension of Chinese and survey content; (4) voluntary participation. In addition, researchers interviewed older adults with disabilities to complete the survey.

### 2.2. Measures

#### 2.2.1. Family function

The Family APGAR Index Questionnaire (APGAR) was used to assess the level of family function among participants (7). Five items, adaptation (utilization of intra- and extra-familial resources for problem-solving when family equilibrium is stressed during a crisis), partnership (sharing of decision-making and nurturing responsibilities among family members), growth (physical and emotional maturation and self-fulfillment achieved by family members through mutual support and guidance), affection (caring or loving relationship between family members) and resolution (commitment to devote time to other family members for physical and emotional nurturing). It also usually involves a decision to share wealth and space), were included in the APGAR questionnaire, and each item received a score of 0–2. The scores of the five questions were summed up to form the total score. A total score of 7–10 indicates good family functioning, while a score of 4–6 and 0–3 indicates moderate and severe family dysfunction, respectively. The Cronbach α coefficient of APGAR in this study was 0.900.

#### 2.2.2. Anxiety

The Generalized Anxiety Scale (GAD-7) was used to assess participants' anxiety symptoms (22). GAD-7 is 7 items and is scored on a 4-point scale with a total score range of 0–21, 0–4 was classified as anxiety without clinical significance, 5–9 as mild anxiety, 10–14 as moderate anxiety, and 15–21 as severe anxiety. Cronbach alpha coefficient of GAD-7 in this study was 0.963.

#### 2.2.3. Depression

The Patient Health Questionnaire Depression Scale (PHQ-9) was used to assess participants' depressive symptoms (23). PHQ-9 is scored on a 4-point scale with a total score range of 0–27, 0–4 is classified as clinically meaningless anxiety, 5–9 is classified as mild depression, 10–14 is classified as moderate depression, 15–19 is classified as moderately severe depression, and 20–27 is classified as severe depression. The Cronbach scale alpha coefficient in this study was 0.947.

### 2.3. Analysis

The analysis was performed with SPSS and R, which analyzed all descriptive statistics and the latter estimates the network structure (24).

#### 2.3.1. Network estimation

According to the recommendations (24), the network model of 23 indicators was estimated using the EBICglasso function in the qgraph package of R. For the network with 23 nodes, 253 parameters [23 × (23–1)/2] need to be estimated (24), and according to at least 3–5 individuals per parameter, the sample size is sufficient for network analysis (*N* = 814). Twemty-three behavioral indicators were depicted as nodes (Table 1), while correlations between symptoms were described as edges in the network. In addition, the mgm package was used to assess the predictability of each node. A node with a high predictability value is indicated by its adjacent nodes, the average predictability of all nodes in a network reflects the extent to which the network is affected by factors outside the network (e.g., environmental and biological factors), and a high average predictability indicates that the network structure is better able to predict each other internally, with less variation explained by external factors (25). In addition, edges can be positive (green lines) or negative (red lines). Stronger connections are represented by thicker and more saturated edges, and nodes with stronger and/or more connections are positioned in closer proximity.

**Table 1:** Mean scores, standard deviations, predictability, and abbreviations for each node of the APGAR, GAD-7, and PHQ-9.

**Node**	**Abbreviation**	***M***	***SD***	**Pre**
Family function (Family APGAR)	/	6.86	3.08	/
APGAR1: adaptation	Adaptation	1.31	0.75	0.54
APGAR2: partnership	Partnership	1.34	0.75	0.67
APGAR3: growth	Growth	1.33	0.73	0.58
APGAR4: affection	Affection	1.38	0.72	0.62
APGAR5: resolve	Resolve	1.50	0.68	0.58
Anxiety symptoms (GAD-7)	/	5.08	6.24	/
GAD1: nervousness or anxiety	Nervousness	0.72	0.95	0.80
GAD2: uncontrollable worry	Uncontrollable worry	0.70	0.99	0.78
GAD3: worry too much	Worry too much	0.81	1.02	0.76
GAD4: relaxing trouble	Relaxing trouble	0.74	0.99	0.79
GAD5: restlessness	Restlessness	0.69	0.96	0.74
GAD6: irritable	Irritable	0.79	1.00	0.79
GAD7: afraid something will happen	Afraid	0.62	0.96	0.73
Depression symptoms (PHQ-9)	/	6.92	7.49	/
PHQ-1: anhedonia	Anhedonia	0.94	1.12	0.60
PHQ-2: depressed or sad mood	Sad mood	0.79	0.99	0.74
PHQ-3: sleep difficulties	Sleep	0.91	1.04	0.71
PHQ-4: feeling tired or having little energy	Energy	0.95	1.03	0.77
PHQ-5: appetite changes	Appetite	0.63	0.88	0.57
PHQ-6: feeling of worthlessness	Worthlessness	0.79	1.01	0.73
PHQ-7: concentration difficulties	Concentration	0.79	1.02	0.67
PHQ-8: Psychomotor agitation/retardation	Motor	0.75	1.00	0.69
PHQ-9: Thoughts on death	Suicide ideation	0.39	0.77	0.56
**Covariates**	/	/	/	/
Gender	Gender	/	/	0.03
Age	Age	/	/	0.03

M, mean; SD, standard deviation; Pre, predictability.

#### 2.3.2. Estimation of centrality and bridge centrality

Centrality measures how directly a node is related to other nodes. Three centrality indices were calculated to assess the importance of each node in the network through the centralityPlot function in the graph package of R (26). The networktools R package was used to calculate bridge centrality statistics, including bridge strength, bridge betweenness and bridge closeness. (1) The overall importance of a symptom in the network is indicated by strength centrality (the sum of weighted values for all connecting lines of a node) (8); (2) closeness centrality (the inverse of the sum of the shortest route distances from other nodes in the network to this node) indicates that the impact of one symptom rapidly spreads to other symptoms (27); (3) betweenness centrality (the frequency of a node on the shortest path of any two other nodes) indicates a bridge symptom connecting with other symptoms and potential target symptoms for intervention (12). The study had shown that the stability of closeness centrality and betweenness centrality is usually low (28), so the standardized strength centrality was primarily reported and based on the standardized bridge strength values of the network, the top 20% scoring nodes were selected as predicted bridge nodes in this study (29).

#### 2.3.3. Network accuracy and stability estimation

The accuracy and stability of the network were calculated using bootstrapping methods in the “bootnet” package (23). First, 95% confidence intervals (CIs) of edge-weight accuracy were calculated by bootstrapping procedures. Then, the stability of the centrality indices was assessed through a case-drop bootstrap, which was evaluated using CS-coefficients (correlation stability). The researchers noted that the CS-coefficient should ideally be above 0.5 but at least above 0.25 (30).

## 3. Results

### 3.1. Participant characteristics

A total of 897 questionnaires were collected online and after removing the same IP address and the number of invalid responses, the valid sample size included 814 (90.00%), with a mean age of 49.17 (ages 18–76, SD = 13.55); 513 male (63%) and 301 female participants (37%); 143 (17.60%) unmarried, 565 (69.40%) married, 71 (8.70%) divorced and 35 (4.30%) widowed; 518 (63.60%) rural and 296 (36.40%) urban residents; and 518 (63.60%) urban residents. The number of participants with 0–6 years of education was 315 (38.70%), 6–9 287 (35.3%), 9–12 103 (12.7%), 12 or more 13 (1.60%), and 96 (11.80%) were uneducated; 338 (41.52%) were working, and 476 (58.48%) were unemployed.

There were 198 (24.30%) congenital disabilities and 616 (75.70%) acquired disabilities. Specifically, 93 (11.40%) have visual disabilities, 51 (6.30%) have hearing disabilities, 482 (29.2%) have physical disabilities, 47 (5.80%) have intellectual disabilities, 19 (2.30%) have speech disabilities, and 122 (122) have multiple disabilities (15.00%). Meanwhile, depression without clinical significance was 408 (50.10%), mild depression 166 (18.30%), moderate depression 99 (12.20%), moderately severe depression 68 (8.40%) and severe depression 73 (9.00%). There were 473 (58.1%) with no clinically significant anxiety, 174 (21.4%) with mild anxiety, 75 (9.20%) with moderate anxiety, and 92 (11.30%) with severe anxiety. In addition, 457 (56.10%) had good family functioning, 230 (28.30%) had moderate family dysfunction, and 127 (15.60%) had severe family dysfunction, Table 2.

**Table 2:** Sample characteristics.

**Variables**	
Age, *M* (*SD*)	49.17(13.55)
**Gender**, ***n*** **(%)**	
Male	513 (63.00%)
Female	301(37.00%)
**Marital status**, ***n*** **(%)**	
Unmarried	143 (17.60%)
Married	565 (69.40%)
Divorced	71 (8.70%)
Widowed	35 (4.30%)
**Education**, ***n*** **(%)**	
Uneducated	96 (11.80%)
0–6 years	315 (38.70%)
6–9 years	287 (35.3%)
9–12 years	103 (12.7%)
12 years or more	13 (1.60%)
**Employment status**, ***n*** **(%)**	
Employed	338 (41.52%)
Unemployed	476 (58.48%)
**Disability type**, ***n*** **(%)**	6.64(3.81)
Congenital disability	175 (23.80%)
Acquired disability	561 (76.20%)
**Depression**, ***n*** **(%)**	
Depression without clinical significance	408 (50.10%)
Mild depression	166 (18.30%)
Moderate depression	99 (12.20%)
Moderately severe depression	68 (8.40%)
Severe depression	73 (9.00%)
**Anxiety**, ***n*** **(%)**	
Anxiety without clinical significance	473 (58.1%)
Mild anxiety	174 (21.4%)
Moderate anxiety	75 (9.20%)
Severe anxiety	92 (11.30%)
**Family function**, ***n*** **(%)**	
Good family function	457 (56.10%)
Moderate family dysfunction	230 (28.30%)
Severe family dysfunction	127 (15.60%)

M, mean; SD, standard deviation; *n*, number of persons.

### 3.2. Network structure

**Figure 1:**
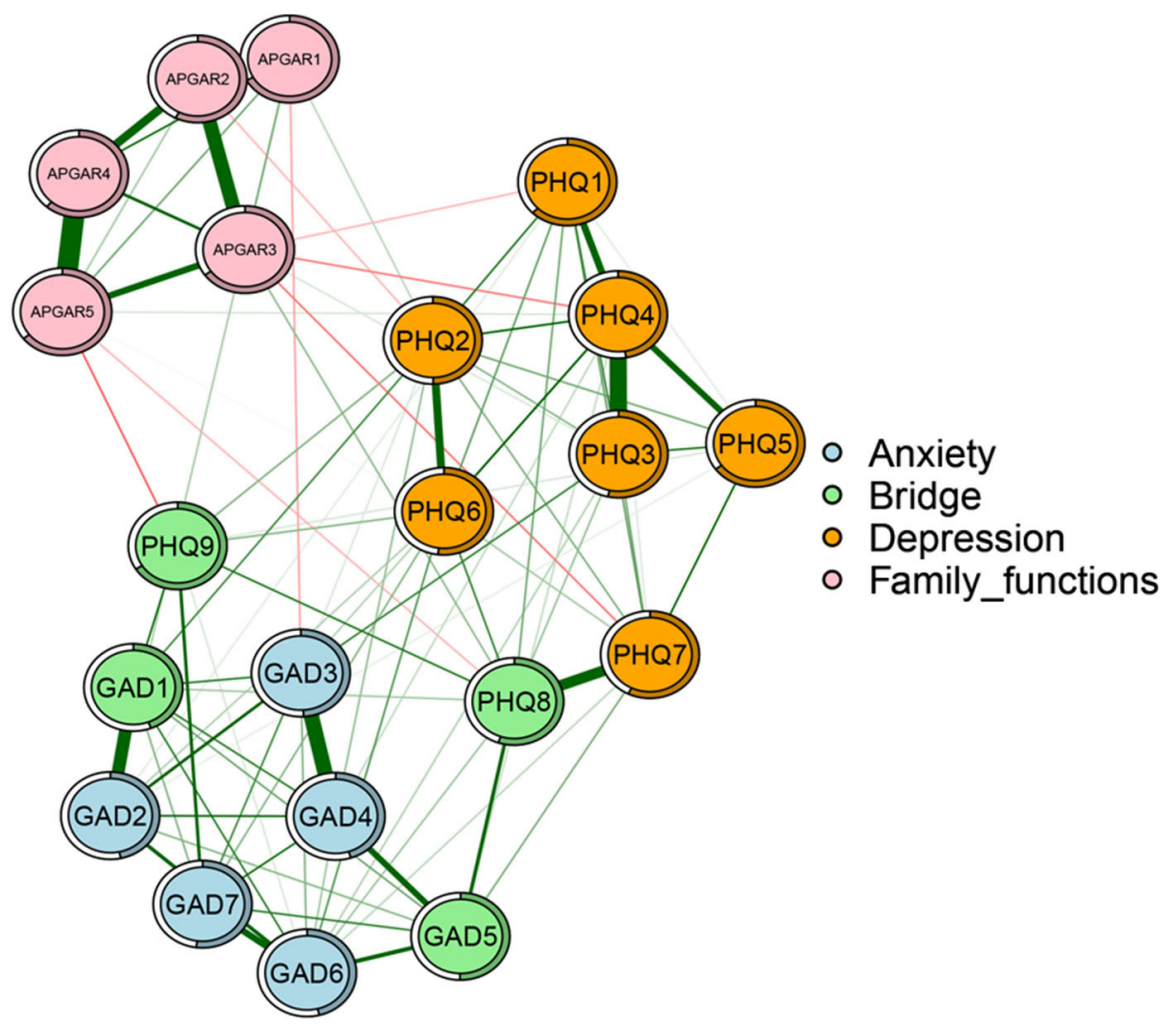
Network of family function, anxiety, and depression nodes in adults with disabilities. Nodes represent family function, anxiety and depression nodes (an identical layout of nodes was imposed), and edges represent partial correlations between symptoms. Edge thickness and darkness indicate association strength (minimum and maximum edge values were set to be equal across networks), and edge color indicates correlation value (green = positive; red = negative). APGAR1, adaptation; APGAR2, partnership; APGAR3, growth; APGAR4, affection; APGAR5, RESOLVE; GAD1, nervousness; GAD2, uncontrollable worry; GAD3, worrying too much; GAD4, trouble relaxing; GAD5, restlessness; GAD6, irritable; GAD7, afraid; PHQ1, anhedonia; PHQ2, sad mood; PHQ3, sleep; PHQ4, energy; PHQ5, appetite; PHQ6, worthlessness; PHQ7, concentration; PHQ8, motor; PHO9, suicide ideation.

The network structure of family function, anxiety and depression among adults with disabilities is illustrated in Figure 1. Ring pie charts of the network were used to indicate node predictability, i.e., mean predictability was 0.69 (range 0.55–0.80, Table 1). With gender and time included as covariates in the network, node APGAR4 (“Affection”) had the most direct connection with node APGAR5 (“Resolve”), followed by the connection among nodes APGAR2 (“Partnership”), APGAR1 (“Adaptation”) and APGAR3 (“Growth”) within the family function community. Among the depressive symptom community, node PHQ4 (“Energy”) had the most direct connection to node PHQ3 (“Sleep”), followed by connection between nodes PHQ7 (“Concentration”) and PHQ8 (“Motor”). Meanwhile, among the anxiety symptom community, node GAD3 (“Worry too much”) had the most direct connection with node GAD4 (“Relaxing Trouble”), followed by the connection between nodes GAD1 (“Nervousness”) and GAD2 (“Uncontrollable Worrying”). In addition, there were many associations among the projects across the three communities. That is, node GAD5 (“Restlessness”) was most strongly associated with node PHQ8 (“Motor”), followed by connections between nodes PHQ9 (“Suicide ideation”) and GAD7 (“Concentration”), and nodes PHQ9 (“Suicide ideation”) and APGAR5 (“Resolve”). In conclusion, node APGAR5 (“Resolve”) directly connects bridge symptoms-PHQ9 (“Suicide ideation”) and PHQ8 (“Motor”).

### 3.3. Centrality and bridge centrality

**Figure 2:**
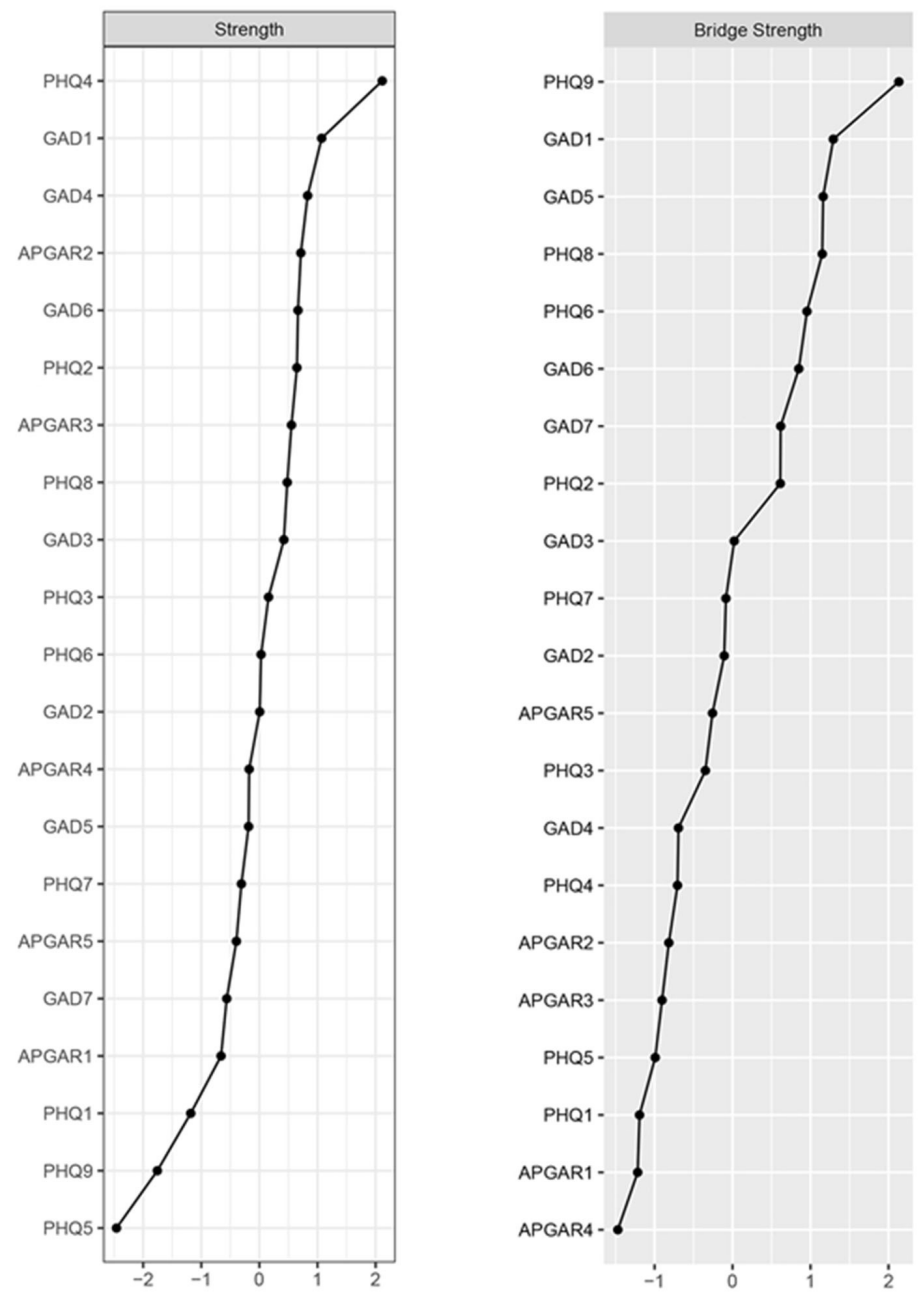
Node strength centrality and bridge strength of the estimated network. Centrality plot depicting the strength centrality of each node in the network (*z*-score); centrality plot depicting the bridge strength centrality of each node in the network (*z*-score). Higher scores represent the node having more influence on the network. APGAR1, adaptation; APGAR2, partnership; APGAR3, growth; APGAR4, affection; APGAR5, resolve; GAD1, nervousness; GAD2, uncontrollable worry; GAD3, worrying too much; GAD4, trouble relaxing; GAD5, restlessness; GAD6, irritable; GAD7, afraid; PHQ1, anhedonia; PHQ2, sad mood; PHQ3, sleep; PHQ4, energy; PHQ5, appetite; PHQ6, worthlessness; PHQ7, concentration; PHQ8, motor; PHO9, suicide ideation.

The centrality and bridge strength of each node among adults with disabilities are shown in Figure 2. Specifically, node PHQ4 (“Energy”) had the highest strength. Nodes APGAR3 (“Growth”), GAD1 (“Nervousness”) and “Relaxing Trouble” (GAD4) were also statistically stronger than most other nodes in the network (Supplementary Figure A1). In terms of bridge strength, nodes PHQ9 (“Suicide ideation”), PHQ6 (“Worthlessness”), GAD1 (“Nervousness”), GAD5 (“Restlessness”) and PHQ8 (“Motor”) were stronger than most other nodes (see Supplementary Figure A2 for other centrality indicators).

### 3.4. Network accuracy and stability

Evaluation of the edge stability indicated moderate stability of the estimated networks: although there were considerable overlaps among 95% of the edge weight CIs, non-overlapped CIs also existed (Supplementary Figure A3) and estimation of the edge weight difference indicated that the higher stability edges were significantly different from other edges in the network (Supplementary Figure A4). Meanwhile, stability estimates for the centrality index showed that the centrality strength stability coefficient (CS-coefficients) was 0.749 (Supplementary Figure A5).

## 4. Discussion

This study used network analysis to construct the network of family function, anxiety and depression symptoms among adults with disabilities. The following are key findings: (1) Nodes PHQ4 (“Energy”), APGAR3 (“Growth”), GAD1 (“Nervousness”) and GAD4 (“Relaxing Trouble”) were central nodes in the network model; (2) Bridge nodes linking family function, anxiety and depressive symptoms in the sample were PHQ9 (“Suicide ideation”), PHQ6 (“Worthlessness”), GAD1 (“Nervousness”) and GAD5 (“Restlessness”); (3) The node APGAR5 (“Resolve”) directly connects the bridge symptoms PHQ9 (“Suicide ideation”) and PHQ8 (“Motor”).

“Energy” (PHQ4) was one of the most central nodes in the family function-anxiety-depression network of adults with disabilities, similar to Fried et al. (2) and Garabiles et al. (31). While previous studies have also shown that anhedonia, depressed mood, low energy, and lack of worthiness are core symptoms (14, 15), inconsistency can be attributed to the type of sample sampling differences. In general, the energy of normal depressed individuals decreases significantly (4), whereas the energy of people with disabilities is lower and they experience greater fatigue or lack of energy as a result of their disabilities and obstacles (3). This may reflect the heterogeneity of depression, but it may also be a comorbid symptom of physical disorders and mental illness. In addition, “Nervousness” (GAD1) and “Relaxing Trouble” (GAD4) were other prominent central symptoms in the family function-anxiety-depression network of adults with disabilities as indicated by its strength. Partly consistent with Wang et al. (32) and Heeren et al. (33) finding trouble relaxing was one of the most central GAD symptoms in a community sample during the 2019 coronavirus disease (COVID-19). Continued localized COVID-19 pandemics and social distancing protocols are prone to symptoms of public anxiety relief and control (34), and data from this study were also collected during this period. In addition, people with disabilities are more sensitive to the environment and their own health due to their limited social functions, so they may have more trouble relaxing and being nervous. This indicated that associations or institutions for the protection of persons with disabilities should pay more attention to the mental health status of persons with disabilities and thus actively and effectively cope with negative emotions during the epidemic.

The “Growth” node (APGAR3), another prominent central node in this network analysis, primarily reflects the support of family members in the process of growth and development of people with disabilities. This may indicate that the support of family members is the key to effective family functioning for preventing and improving depression among people with disabilities. The current study also shows that family support is effective in increasing life satisfaction and decreasing depression during the disabled person's own development. Sachs-Ericsson et al. (35) also found an association between family dysfunction and suicidal behavior in patients with psychiatric disorders. Family systems theory (7) believes that families are composed of several subsystems that support each other to effectively perform the function of the family system and promote the positive interaction and wellbeing of family members. This also may indicate that the positive cooperation of family members is the key to the effective realization of family function. Therefore, therapists should fully raise the awareness of family members, build positive and mutually supportive relationships, and promote the healthy development of the family system and thus alleviate individual mental disorders in the process of future family therapy.

Meanwhile, this study showed that “Suicide ideation” (PHQ9), PHQ6 (“Worthlessness”), “Nervousness” (GAD1) and “Restlessness” (GAD5) were bridge symptoms in this network. “Suicide ideation” (PHQ9), an important clinical feature of depression, was the most common symptom of bridge strength. A recent meta-analysis indicated that people with somatic disorders are more likely to have suicidal thoughts and commit suicide (36), especially when depression is also present (37). The hopelessness theory of depression (9) suggests that adverse external stimuli (e.g., disability) are prone to induce despair, helplessness, and uselessness in individuals, resulting in thoughts of ending life and escaping pain, and a more tolerant and accepting attitude toward suicide. Thus, people with disabilities with some degree of functional impairment may have thoughts of death if they also have other recurrent depressive symptoms. In addition, studies have also revealed that both suicide and suicidal ideation are associated with dysfunction in inflammatory, immune, and stress response systems, which partially underlie disability (38). In addition, the findings are aligned with previous reports in which “Worthlessness” (PHQ6), “Nervousness” (GAD1), and “Restlessness” (GAD5) were bridge symptoms between depression and anxiety among different groups. In general, there is a significant decrease in energy in depressed patients without physical disabilities (2), which is exacerbated by the physical impairments and partial loss of social functioning of people with disabilities, who are more prone to nervousness, restlessness, and feelings of worthlessness (39). Thus, nodes “Worthlessness” (PHQ6), “Nervousness” (GAD1) and “Restlessness” (GAD5) may be important channels through which depression and anxiety symptoms interact and play the most prominent role in activating and sustaining the psychopathological network of depression and anxiety.

Furthermore, the network on the effects of family function on depression and anxiety revealed that bridging symptoms PHQ9 (“Suicide ideation”) and PHQ8 (“Motor”) were important in connecting family function (Resolve: APGAR 5) with depression and anxiety symptoms. This suggests that the resolve of family members may be an important factor influencing anxiety and depression in people with disabilities. Meanwhile, the general strain theory (21) indicates that nervousness caused by a lack of interpersonal relationships makes individuals prone to psychological and behavioral disorders. Social support provided by positive interpersonal relationships could relieve stress and alleviate negative emotions. Therefore, while activation of bridge symptoms may increase the risk of disorder through activation of other symptoms by the resulting symptom, targeted interventions for bridge components may prevent the development of clinical disorders (16, 29). These indicated therapists could target the resolve of family members during family therapy to reduce suicidal ideation and enhance the level of activity of people with disabilities, thereby improving the network of anxiety and depression symptoms and alleviating negative emotions of people with disabilities.

In addition, although this study has some clinical implications, there are some limitations: (1) This study explored the relationship between family function, anxiety, and depressive symptoms from a cross-sectional perspective through network analysis, which has some clinical implications for improving anxiety and depression in people with disabilities through family therapy. However, cross-sectional data evidence is limited, so future studies should analyze their dynamic relationships based on longitudinal data to guide clinical practice with more robust and rigorous findings; (2) The disability population included in this study were all adults who volunteered to participate and the sample size was adequate for statistical analysis but not a large sample, so there may be some selection bias and therefore caution should be taken in generalizing the findings; (3) The study found a strong association between resolve of family functioning, anxiety and depressive symptoms among people with disabilities, but further clinical research is needed to confirm that family therapy improves the resolve of family members, which in turn alleviates anxiety and depressive symptoms among people with disabilities. (4) Although this study suggests that researchers should focus on the physical and mental health of the disabled from the family system's perspective and its needs. Simultaneously, because of the inconsistency in the needs of different types of persons with disabilities, future research should also consider the impact of the specificity of individuals with different types of disabilities.

In conclusion, the study suggested that therapists could target the resolve of family members during family therapy to reduce suicidal ideation and enhance the level of activity of people with disabilities, thereby improving the network of anxiety and depression symptoms and alleviating negative emotions of people with disabilities.

## Data availability statement

The raw data supporting the conclusions of this article will be made available by the authors, without undue reservation.

## Ethics statement

The project has passed the ethical review of the Academic Committee of the College of Law of Southwest University of Science and Technology, and the ethical review approval number is: LL23001. The studies were conducted in accordance with the local legislation and institutional requirements. Written informed consent for participation in this study was provided by the participants' legal guardians/next of kin. Written informed consent was obtained from the individual(s) for the publication of any potentially identifiable images or data included in this article.

## Author contributions

DY and BW: conception and design. XZ and HF: provision of study materials. XZ, FY, and BW: collection and assembly of data. XZ, FY, HF, and BW: data analysis and interpretation. DY, BW, and HF: manuscript writing. All authors contributed to the article and approved the submitted version.

## Footnotes

^1^Disability. Available online at: https://www.who.int/health-topics/disability (accessed September 24, 2023).

## References

[1] Liu L Zhang Y. Relationship between stigma and mental health of physicaly disabled: mediating effect of resilience. Psychiatr Danub. (2021) 33:560–5. 10.24869/psyd.2021.560 34928904

[2] Fried EI Epskamp S Nesse RM Tuerlinckx F Borsboom D. What are “good” depression symptoms? Comparing the centrality of DSM and non-DSM symptoms of depression in a network analysis. J Affect Disord. (2016) 189:314–20. 10.1016/j.jad.2015.09.005 26458184

[3] Lamers F van Oppen P Comijs HC Smit JH Spinhoven P van Balkom AJLM. Comorbidity patterns of anxiety and depressive disorders in a large cohort study: the Netherlands Study of Depression and Anxiety (NESDA). J Clin Psychiatry. (2011) 72:341–8. 10.4088/JCP.10m06176blu 21294994

[4] Jilili M Liu L. Examining the impact of functional disability and cognitive impairment on mental health of Chinese elderly. Soc Work Health Care. (2022) 61:338–52. 10.1080/00981389.2022.2091080 35792711

[5] Glombiewski JA Hartwich-Tersek J Rief W. Two psychological interventions are effective in severely disabled, chronic back pain patients: a randomised controlled trial. Int J Behav Med. (2010) 17:97–107. 10.1007/s12529-009-9070-4 19967572

[6] Marti CN Kunik ME Choi NG. The reciprocal relationship between depression and disability in low-income homebound older adults following tele-depression treatment. Int J Geriatr Psychiatry. (2021) 36:802–10. 10.1002/gps.5480 33275787 PMC8855885

[7] Smilkstein G. The family APGAR: a proposal for a family function test and its use by physicians. J Fam Pract. (1978) 6:9. 660126

[8] Miller IW Ryan CE Keitner GI Bishop DS Epstein NB. The McMaster Approach to Families: theory, assessment, treatment and research. J Fam Ther. (2000) 22:168–89. 10.1111/1467-6427.00145

[9] Abramson LY Metalsky GI Alloy LB. Hopelessness depression: a theory-based subtype of depression. Psychol Rev. (1989) 96:358–72. 10.1037/0033-295X.96.2.358

[10] McNally RJ Robinaugh DJ Wu GWY Wang L Deserno MK Borsboom D. Mental disorders as causal systems: a network approach to posttraumatic stress disorder. Clin Psychol Sci. (2015) 3:836–49. 10.1177/2167702614553230

[11] Bringmann LF Lemmens LHJM Huibers MJH Borsboom D Tuerlinckx F. Revealing the dynamic network structure of the Beck Depression Inventory-II. Psychol Med. (2015) 45:747–57. 10.1017/S0033291714001809 25191855

[12] Borsboom D. A network theory of mental disorders. World Psychiatry. (2017) 16:5–13. 10.1002/wps.20375 28127906 PMC5269502

[13] Borsboom D Cramer AOJ. Network analysis: an integrative approach to the structure of psychopathology. Annu Rev Clin Psychol. (2013) 9:91–121. 10.1146/annurev-clinpsy-050212-185608 23537483

[14] McNally RJ. Can network analysis transform psychopathology? Behav Res Ther. (2016) 86:95–104. 10.1016/j.brat.2016.06.006 27424882

[15] Bryant RA Creamer M O'Donnell M Forbes D McFarlane AC Silove D. Acute and chronic posttraumatic stress symptoms in the emergence of posttraumatic stress disorder: a network analysis. JAMA Psychiatry. (2017) 74:135. 10.1001/jamapsychiatry.2016.3470 28002832

[16] Makhubela M. Comorbid anxiety and depression psychopathology in university students: a network approach. South Afr J Psychol. (2021) 51:35–53. 10.1177/0081246320973839

[17] Briganti G Scutari M Linkowski P. Network structures of symptoms from the zung depression scale. Psychol Rep. (2021) 124:1897–911. 10.1177/0033294120942116 32686585

[18] Beard C Millner AJ Forgeard MJC Fried EI Hsu KJ Treadway MT. Network analysis of depression and anxiety symptom relationships in a psychiatric sample. Psychol Med. (2016) 46:3359–69. 10.1017/S0033291716002300 27623748 PMC5430082

[19] Zhang P Wang L Zhou Q Dong X Guo Y Wang P. Network analysis of anxiety and depression symptoms in Chinese disabled elderly. J Affect Disord. (2023) 333:535–42. 10.1016/j.jad.2023.04.065 37086797

[20] Bertelli M Bianco A Rossi M Scuticchio D Brown I. Relationship between individual quality of life and family quality of life for people with intellectual disability living in Italy. J Intellect Disabil Res. (2011) 55:1136–50. 10.1111/j.1365-2788.2011.01464.x 21883597

[21] Agnew R. Foundation for a general strain theory of crime and delinquency^*^. Criminology. (1992) 30:47–88. 10.1111/j.1745-9125.1992.tb01093.x

[22] Esser P Hartung TJ Friedrich M Johansen C Wittchen H-U Faller H. The Generalized Anxiety Disorder Screener (GAD-7) and the anxiety module of the Hospital and Depression Scale (HADS-A) as screening tools for generalized anxiety disorder among cancer patients. Psychooncology. (2018) 27:1509–16. 10.1002/pon.4681 29473255

[23] Tomitaka S Kawasaki Y Ide K Akutagawa M Yamada H Ono Y. Distributional patterns of item responses and total scores on the PHQ-9 in the general population: data from the National Health and Nutrition Examination Survey. BMC Psychiatry. (2018) 18:1–9. 10.1186/s12888-018-1696-9 29685128 PMC5913886

[24] Epskamp S Borsboom D Fried EI. Estimating psychological networks and their accuracy: a tutorial paper. Behav Res Methods. (2018) 50:195–212. 10.3758/s13428-017-0862-1 28342071 PMC5809547

[25] Liu X Wang H Zhu Z Zhang L Cao J Zhang L. Exploring bridge symptoms in HIV-positive people with comorbid depressive and anxiety disorders. BMC Psychiatry. (2022) 22:1–10. 10.1186/s12888-022-04088-7 35790936 PMC9254609

[26] Opsahl T Agneessens F Skvoretz J. Node centrality in weighted networks: generalizing degree and shortest paths. Soc Netw. (2010) 32:245–51. 10.1016/j.socnet.2010.03.006

[27] Borgatti SP Mehra A Brass DJ Labianca G. Network analysis in the social sciences. Science. (2009) 323:892–5. 10.1126/science.1165821 19213908

[28] Birkeland MS Greene T Spiller TR. The network approach to posttraumatic stress disorder: a systematic review. Eur J Psychotraumatology. (2020) 11:1700614. 10.1080/20008198.2019.1700614 PMC6968637 32002135

[29] Jones PJ Ma R McNally RJ. Bridge centrality: a network approach to understanding comorbidity. Multivar Behav Res. (2021) 56:353–67. 10.1080/00273171.2019.1614898 31179765

[30] Armour C Fried EI Deserno MK Tsai J Pietrzak RH. A network analysis of DSM-5 posttraumatic stress disorder symptoms and correlates in US military veterans. J Anxiety Disord. (2017) 45:49–59. 10.1016/j.janxdis.2016.11.008 27936411

[31] Garabiles MR Lao CK Xiong Y Hall BJ. Exploring comorbidity between anxiety and depression among migrant Filipino domestic workers: a network approach. J Affect Disord. (2019) 250:85–93. 10.1016/j.jad.2019.02.062 30836284

[32] Wang Y Hu Z Feng Y Wilson A Chen R. Changes in network centrality of psychopathology symptoms between the COVID-19 outbreak and after peak. Mol Psychiatry. (2020) 25:3140–9. 10.1038/s41380-020-00881-6 32929212 PMC7488637

[33] Heeren A Hanseeuw B Cougnon L-A Lits G. Excessive worrying as a central feature of anxiety during the first COVID-19 lockdown-phase in Belgium: insights from a network approach. Psychol Belg. (2021) 61:401–18. 10.5334/pb.1069 35070347 PMC8719470

[34] Hoffart A Johnson SU Ebrahimi OV. The network of stress-related states and depression and anxiety symptoms during the COVID-19 lockdown. J Affect Disord. (2021) 294:671–8. 10.1016/j.jad.2021.07.019 34333175 PMC8433602

[35] Sachs-Ericsson NJ Stanley IH Sheffler JL Selby E Joiner TE. Non-violent and violent forms of childhood abuse in the prediction of suicide attempts: Direct or indirect effects through psychiatric disorders? J Affect Disord. (2017) 215:15–22. 10.1016/j.jad.2017.03.030 28292658

[36] Xiong F Wang L Shen L Guo W Li S Guan Q. The relationship between multimorbidity and suicidal ideation: a meta-analysis. J Psychosom Res. (2020) 138:110257. 10.1016/j.jpsychores.2020.110257 32992210

[37] Webb RT Kontopantelis E Doran T Qin P Creed F Kapur N. Suicide risk in primary care patients with major physical diseases: a case-control study. Arch Gen Psychiatry. (2012) 69:256–64. 10.1001/archgenpsychiatry.2011.1561 22393218

[38] Turecki G Brent DA Gunnell D O'Connor RC Oquendo MA Pirkis J. Suicide and suicide risk. Nat Rev Dis Primer. (2019) 5:1–22. 10.1038/s41572-019-0121-0 31649257

[39] Shandra CL Kruger A Hale L. Disability and sleep duration: evidence from the American Time Use Survey. Disabil Health J. (2014) 7:325–34. 10.1016/j.dhjo.2014.02.002 24947574

