# Supplementary material for: Family function, anxiety and depression in adults with disabilities: a network analysis
Source: Front Public Health. 2023 Oct 31;11:1181203. doi: 10.3389/fpubh.2023.1181203 (PMC10644035; doi:10.3389/fpubh.2023.1181203)
Supplement: Supplementary file 1 [file Data_Sheet_1.docx]

**Supplementary**

**
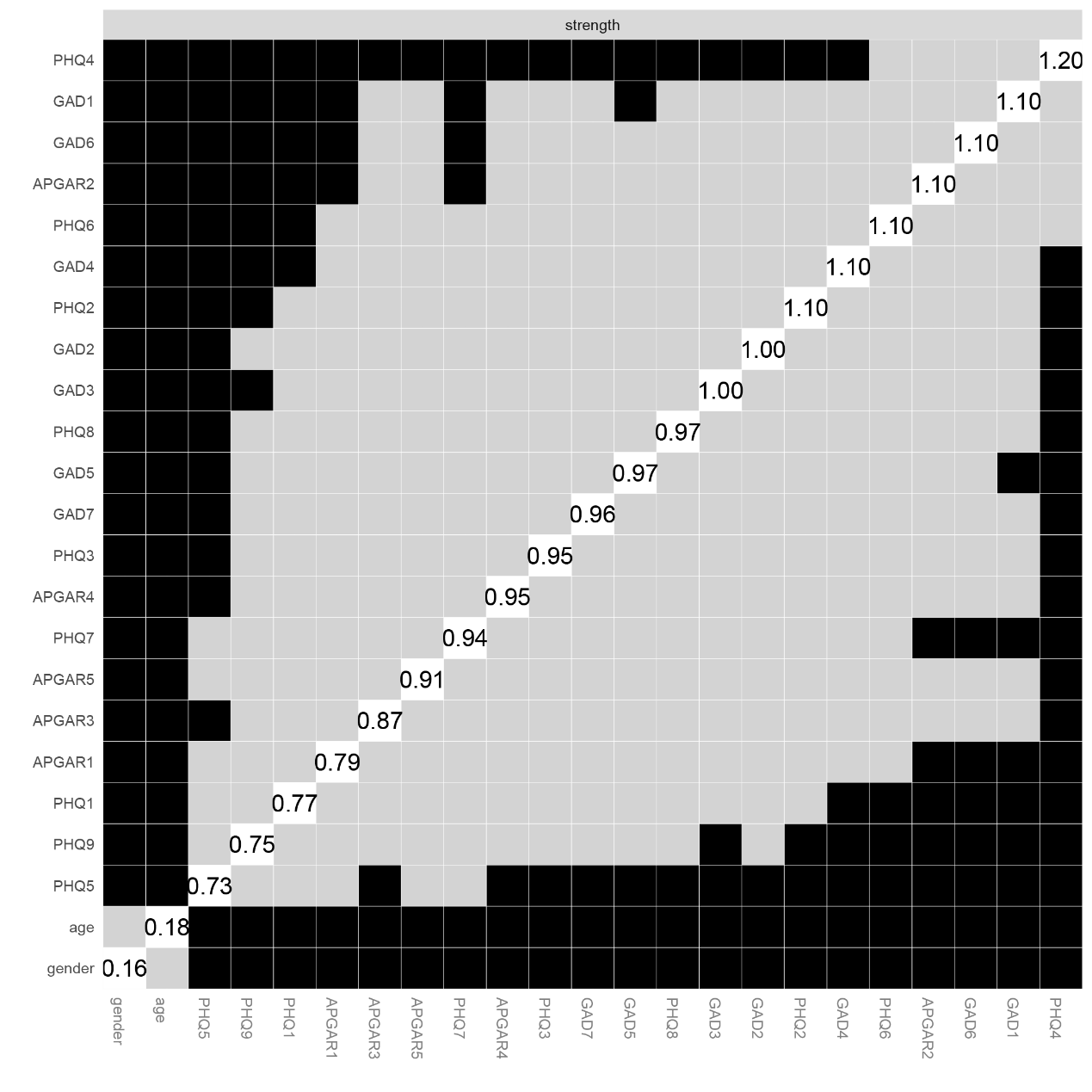
Figure S1** **Estimation of node strength difference by bootstrapped difference test.** Bootstrapped difference tests between node strength of factors. Gray boxes indicate nodes that do not significantly differ from one-another. Black boxes represent nodes that significantly differ from one another (α = 0.05). White boxes show the values of node strength.

**Figure S2 Node centrality and bridge centrality of the estimated network.** Centrality plot depicting the strength centrality of each node in the network (z-score); centrality plot depicting the bridge strength centrality of each node in the network (z-score). Higher scores represent the node having more influence on the network. APGAR1: Adaptation; APGAR2: Partnership; APGAR3: Growth; APGAR4: Affection; APGAR5: Resolve; GAD1, Nervousness; GAD2, Uncontrollable worry; GAD3, Worrying too much; GAD4, Trouble relaxing; GAD5, Restlessness; GAD6, Irritable; GAD7, Afraid; PHQ1, Anhedonia; PHQ2, Sad mood; PHQ3, Sleep; PHQ4, Energy; PHQ5, Appetite; PHQ6, Worthlessness; PHQ7, Concentration; PHQ8, Motor; PHO9 Suicide ideation

**
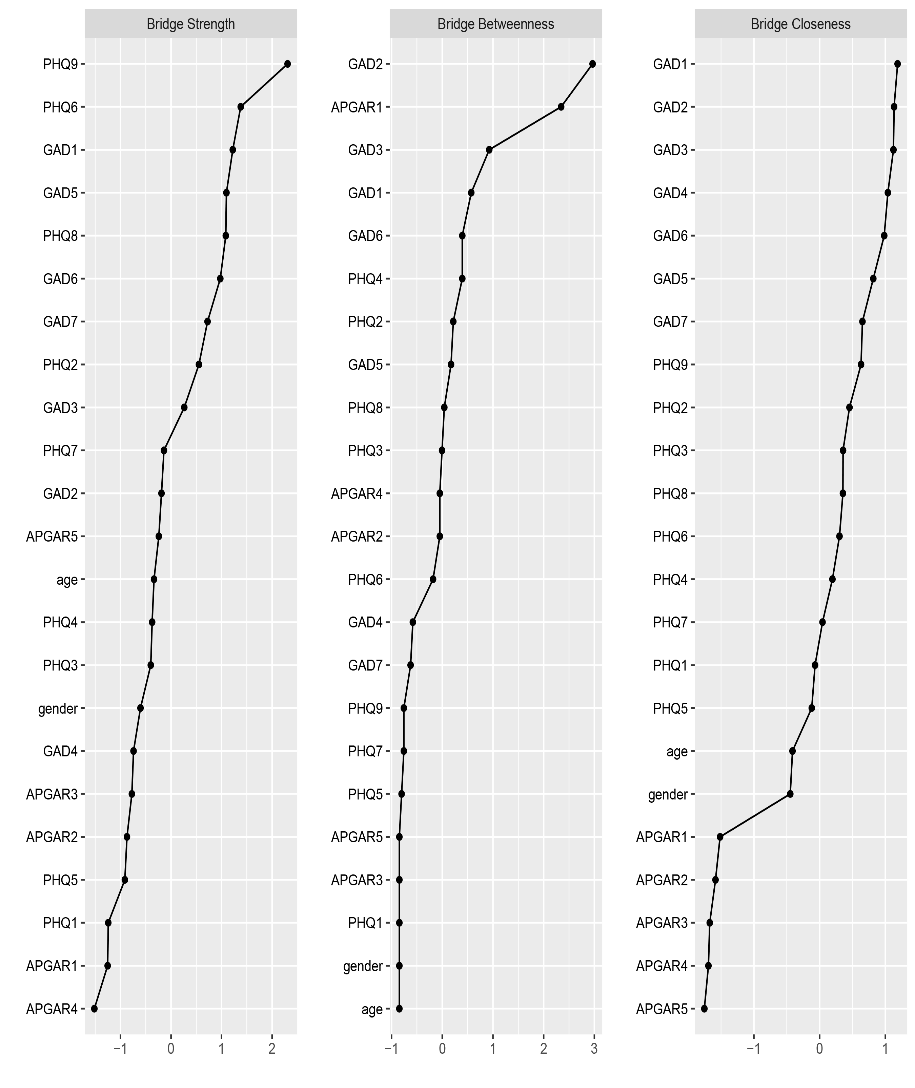

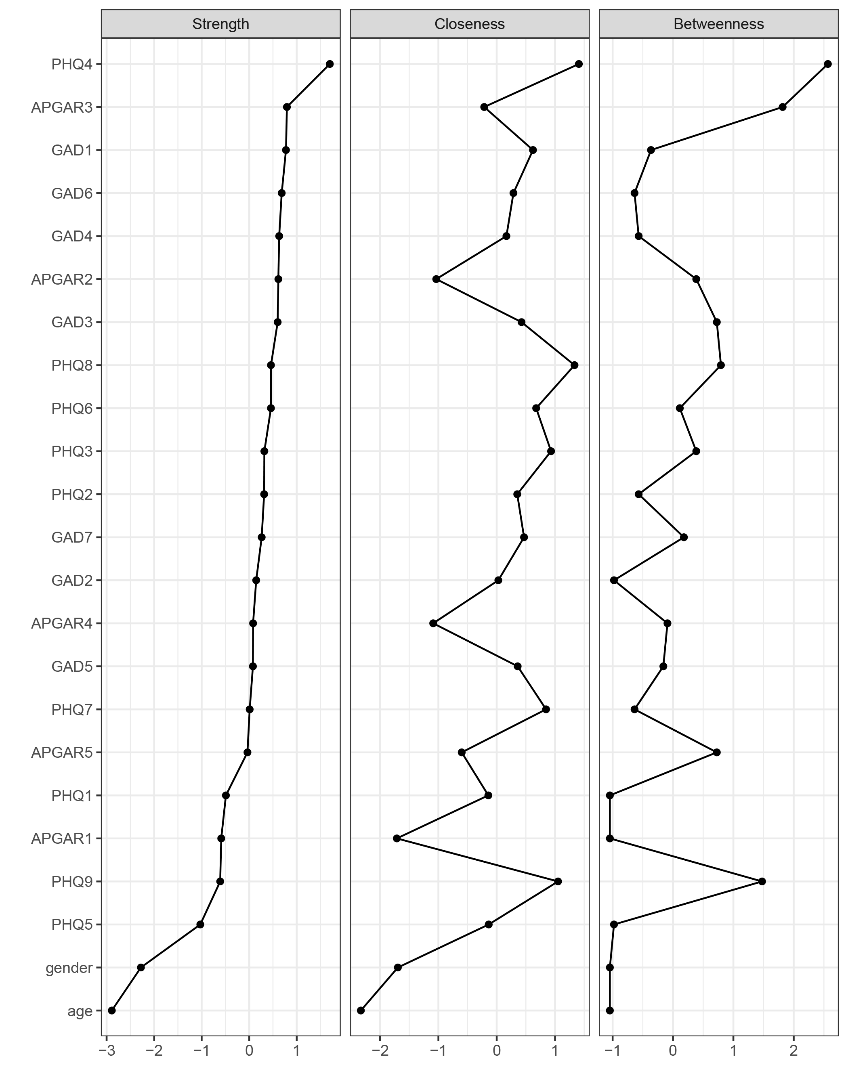
**

**Figure S3 Accuracy of the edge-weights for the current network model.** The gray area represents 95% Confidence Intervals of edge weights, estimated with the non-parametric bootstrap procedure. Wide intervals indicate lower stability and narrow intervals indicate higher stability. The red dots indicate sample values, while the black dots indicate the values of each edge weight, ordered from highest to lowest.

**
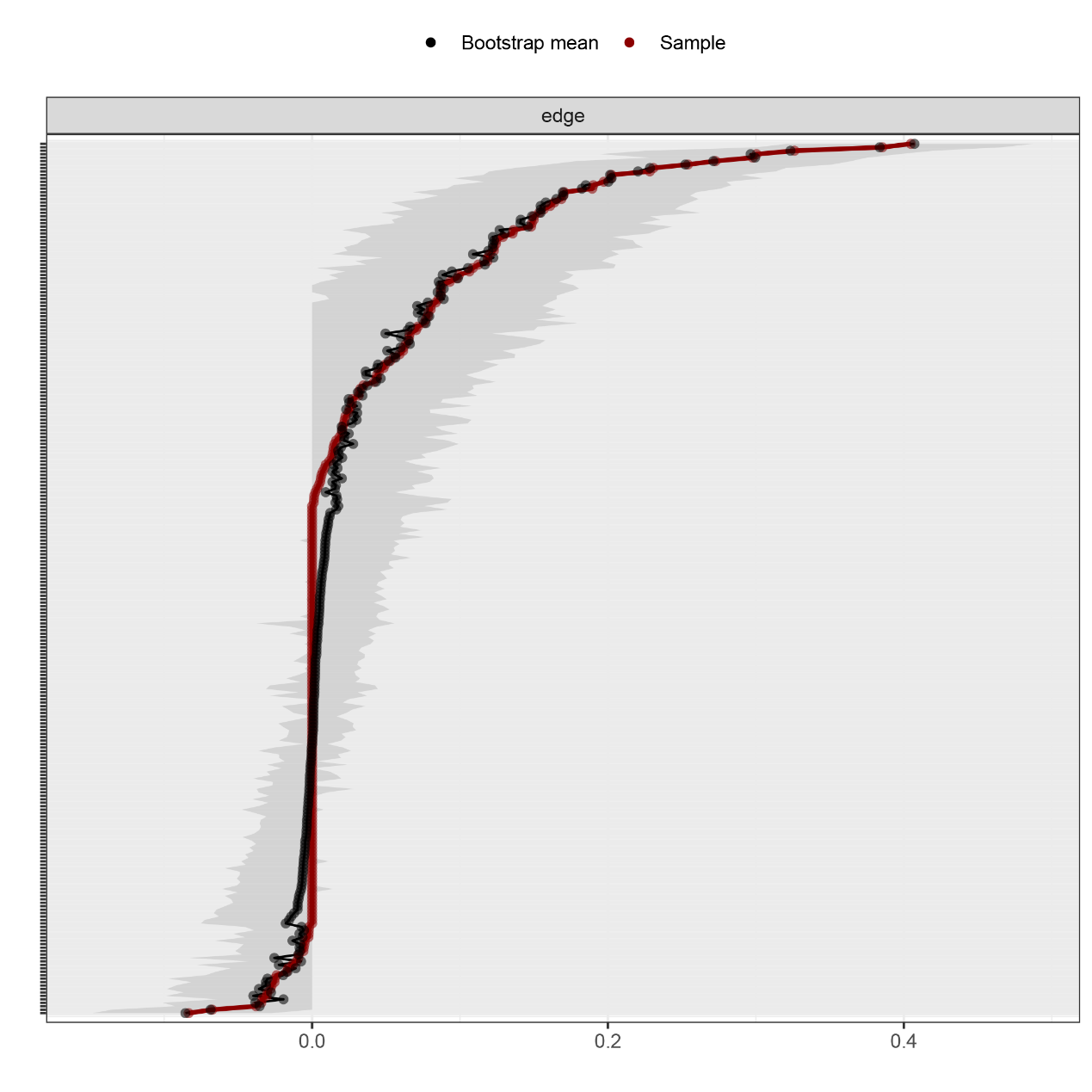
**

**Figure S4 Estimation of edge weight difference by bootstrapped difference test.** Bootstrapped difference tests between edge weights in the network. Gray boxes indicate edges that do not significantly differ from one-another. Black boxes represent edges with significant difference from one another (α = 0.05). Blue boxes in the edge-weight plot indicate positive correlations, and orange boxes in the edge-weight plot indicate negative correlations.


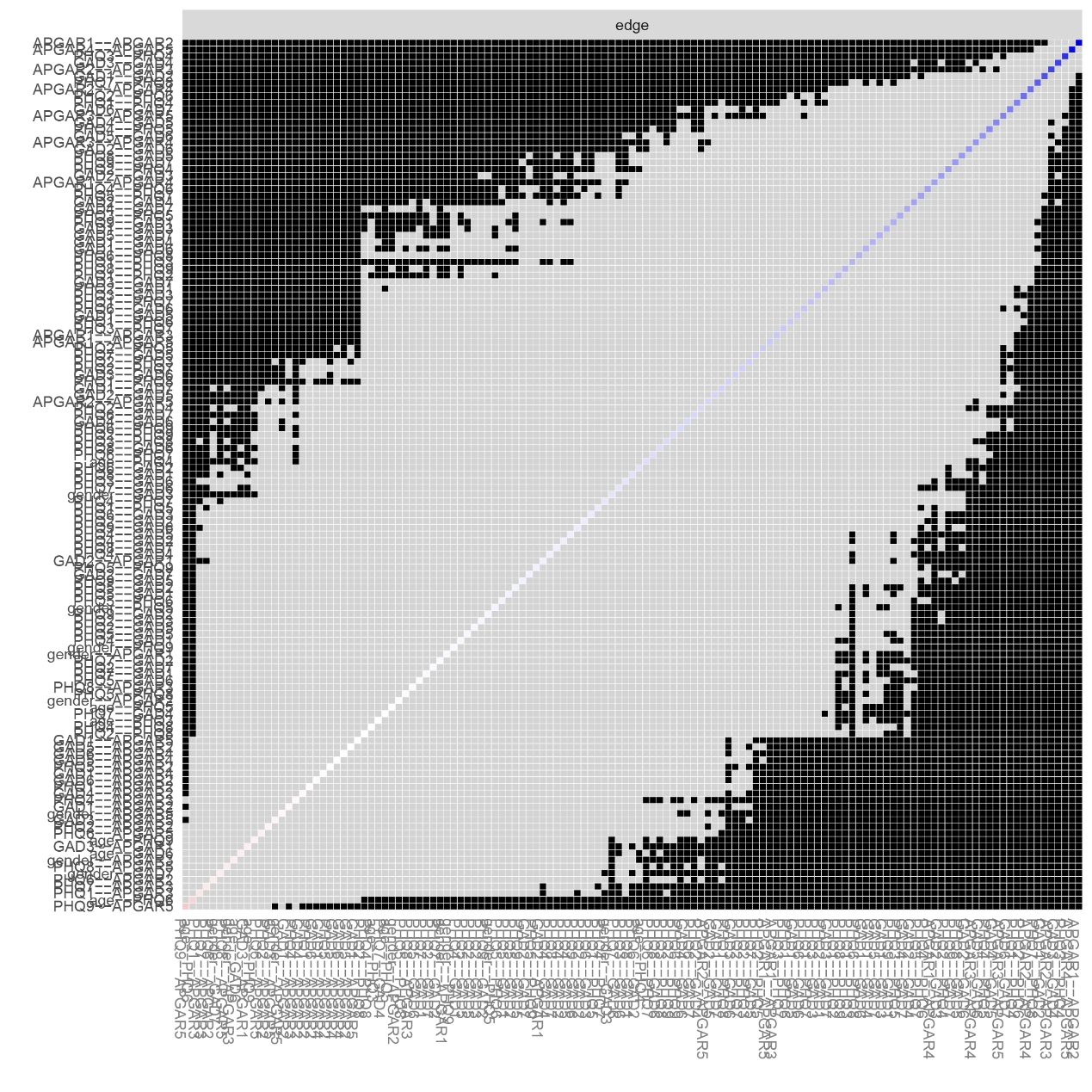


**Figure S5 Stability of centrality indices by case dropping subset bootstrap.** The x axis represents the percentage of cases of original sample used at each step. The y axis represents the average of correlations between the centrality indices from the original network and the centrality indices from the networks that were re-estimated after dropping increasing percentages of cases. Each line indicates the correlations of strength, while areas indicate 95% CI.

**
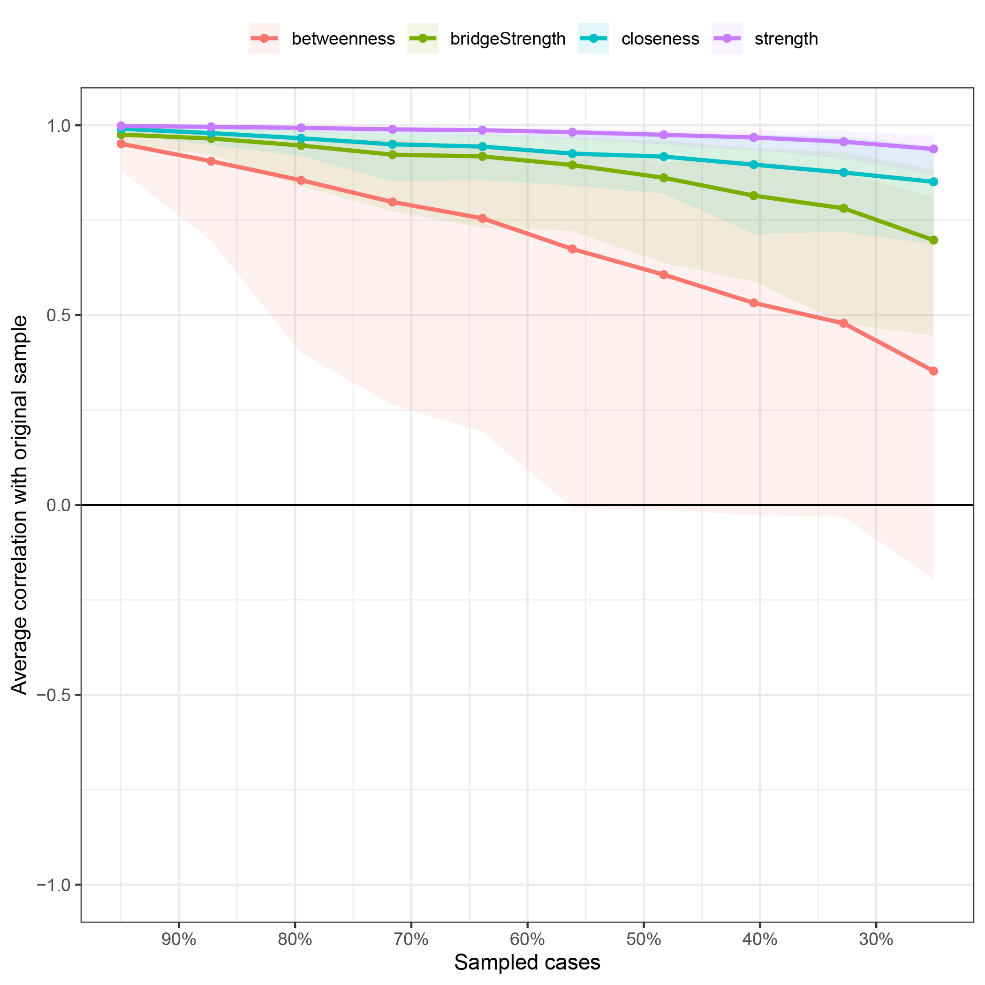
**

Note:

betweenness: 0.128

bridge strength: 0.517

closeness: 0.749

strength: 0.749
